# Moral Insanity—What Is It?*A paper read at a meeting of the Toronto Medical Society, December 14, 1882.

**Published:** 1883-11

**Authors:** J. Workman

**Affiliations:** Toronto; Late Superintendent of the Toronto Asylum for the Insane, etc.


MORAL INSANITY—WHAT IS IT?* By J. WORKMAN, M.D., TORONTO. Late Superintendent of the Toronto Asylum for the Insane, etc.

In the whole range of medical literature there is, perhaps, no nosological term which has been provocative of keener criticism within the ranks of alienistic specialists, and certainly none which has invited more of vapid pedantic invective, in the outside world, whether at the bar, or in the bar room, on the bench, or in blue stocking sewing circles, than the hapless misnamed creature which I have selected for your present merciful consideration. I would trust that as the untimely born thing had no voice in its own baptismal designation, but has owed the infliction to one of our own countrymen, who was dissatisfied with the names previously given to it by a couple of Frenchmen, too recently before, and too soon after the battle of Waterloo, you will approach the subject with becoming moderation, and as far as in you may lie, endeavor to rescue it from the approbrium under which it has innocently suffered.

In the year of 1835 Dr. Pritchard gave to the British public his very valuable treatise on insanity. Nothing can be more unfortunate than the enunciation, in any department of science, of an erroneous doctrine by an author of celebrity. The world has suffered enormous evils, resulting from this source; and I fear not a few of its insane portion have been subjected to very unjust and very unwise judicial penalties, as the direct outcome of Dr. Pritchard's indiscreet nomenclature. Had he been content with the simple depiction of cases of unquestionable mental alienation, so graphically, and I doubt not, so faithfully recorded by him, in which the prominent and high overtopping manifestation of mental alienation, was that of moral abnormality, or utter disregard of the proprieties and conventionalities of social life, his work might well command enduring veneration and gratitude; but he

*A paper read at a meeting of the Toronto Medical Society, December 14, 1882.

had the misfortune to introduce into the science of alienism that ill-fated term—*moral insanity*—as significant of a form of the disease in which the intellect continues quite intact. It is not outside the range of the possibilities, that, had he dubbed his new birth by some different name, and insisted less pertinaciously on its specificity, his theory might have had a longer run of uncontroverted domination. I think, had he consulted me, I would have advised him to call it *Insane Morality*, and I think any discerning reader who will calmly and and carefully analyse the seventeen cases given by him as illustrations of the malady would admit the desirability of the change. But what is the use of writing a book that introduces nothing of novelty? Pinel, in 1809, treated of a form of insanity apparently very similar to, if not identical with Pritchard's Moral Insanity. He called it *manie sans délire.* His pupil, Esquirol, following docilely in the footprints of his venerated master, expanded the subject, and in one of the best works yet devoted to the subject of mental disease, he has given several examples of the same affection, but like Pritchard, he tries his hand in the nominating line of business. Pinel's Madness without Delirium and Pritchard's Moral Insanity, become under his sponsorship *manie raisonnante*—reasoning madness—a designation which to ninety-nine and more of every hundred of the uninitiated, who have been taught to believe that the insane always rave and never reason, could not fail to appear utterly absurd. Hence, no doubt, Pinel and Esquirol, and all who have ever expressed concurrence in their theory, have been regarded as themselves insane, and have been much pitied as victims, by contagion to the dreadful malady, to the practical study and amelioration of which they had too earnestly devoted their time and their untiring efforts.

Yet gentlemen, there is much valuable instruction in the works of Pinel, Esquirol and Pritchard to all such as will sedulously distil it out. The insane do not always rave, nor do those who rave, always do so; the insane sometimes reason, occasionally indeed, a little too sharply as I have often known for those who address them as if taking them for mindless bipeds; and I apprehend it is within the knowledge of most of us that the morality of the insane is not always of unexceptionable purity.

Every man must, from his own consciousness, feel convinced that the human mind, or if I may without offense use the term, the human soul, embraces in its domain something more than mere intellect. We all *feel* as well as *think*, and our judgment is often influenced by our feelings; in too many instances, indeed, the latter obscure or warp or even completely subjugate the former. It is a great error to cut the mind up into distinct and independent principalities, any one of which may pass into a state of rebellion or anarchy, without disturbing the peace or even endangering the normal integrity of others. Those who have had sufficient opportunities of observing the primary manifestations of mental disease, must be able to testify, that in very many instances long before any disorder or impairment of the intellect has been noticed or detected, some unaccountable change has been exhibited in the feelings, the moral sentiments, or the conduct and social demeanor of its destined victims.

The temper which, erewhile, was mild, equable and cheerful, has become irritable, changeable, morose, or perhaps extravagantly joyous. The loving husband has become harsh and tyrannous, the tender parent has become capriciously cruel to the children once the objects of his intense love, the happy home has been transformed into a den of perpetual misery, strife, recrimination, and, but too frequently, acts of dangerous violence. It is needless to amplify the picture. Materials for the filling up may be found in many an unhappy household. Within the last two years a case came to my knowledge strikingly illustrative of the fact which I here desire to accentuate. The subject of it was, till within a few months prior, an intelligent, industrious, good-living man. In consequence of failing in business, he became gloomy, taciturn, and utterly despondent. He continued in this state for some weeks, but under the kind and judicious care of a devoted and sensible wife, improvement gradually took place, and his former mental composure returned. Meeting with a chance of embarking in a line of business suited to his capacity, and very restorative to his exhausted purse, he became very energetic, and as fertile in speech as he before had been reticent. He resided not far from me, and I watched him with solicitude. I feared that he would bear his prosperity no better than he had done his adversity. My fears were too fully justified. He embarked in a very problematic business enterprise, despite the advice of his wife and all his best friends; his temper became very irritable and at times ominously violent. His wife and the children were forced to leave him. Their religious pastor approved of the precaution, and after hearing full details, I advised her not to venture back until a promising change is apparent.

Now, what was the mental condition of this poor man? So far as his intellect was concerned, no outsider coming to do business, or to converse with him, could detect any flaw or impairment, and I believe it would be impossible for any three medical practitioners undertaking examination of his mental state to find in his conversation, or his deportment towards them, adequate facts to enable them to fill up the first question required to be answered in the statutory certificate of lunacy, which is indispensable to the commitment of a person to asylum custody. Should he commit some capital offense, every judge, jury, or crown prosecutor, that I have yet encountered, would pooh pooh the idea of his insanity; the newspaper reporters would, in due course, have to record of him, *abiit ad plures.* And yet, gentleman, this man's case is exactly one of that class which Pritchard, Ray, and other illustrious writers have ventured to call *moral insanity*; but woe and abiding ridicule betide the medical witness who might, when pushed by an ardent prosecutor to mention the *class* of insanity in which he would place the case, be so indiscreet as to utter this term! He would be sure to hear the prisoner ordered to be *corded* up, and he would feel inclined to try the experiment on himself as his only escape from the tortures awaiting him at the hands of a few who bid fair for yet passing a satisfactory examination for asylum honors.*

It is indeed high time that the obnoxious, death-bearing words should be expunged from the literature of alienism and from the minds of the whole community. It is my belief that the term

* We understand that this person has another attack of the same character.—EDS.

is as unhappy as it is uncalled for. An extended and close *practical* study of insanity in its every phase and degree, from its earliest inception up to its final culmination, would, I think, convince every careful observer that the line of demarcation drawn by some writers between what they have called moral insanity and unsoundness of the intellect, is often very untraceable, and that lapse of time and intimate observance are all that is necessary for its total obliteration. Of seventeen cases of moral insanity recorded by Prichard, hardly one, I think, will stand the test of critical analysis. They were all more or less dashed with streaks of actual impairment of intellect, or the majority of them ran on into undeniable mental overthrow; one, indeed, turned out to be a case of a form of intellectual wreck, which, at the present day, no asylum physician could fail to recognize as appertaining to the most hopeless and the most salient of all the forms of mental dethronement. I refer to general paresis,—a form of brain disease which would appear to have been but little prevalent, or little known, fifty years ago, but which, in the present day, seems to be making deathly strides, even in our sparsely peopled province.

Those who throw their aspersions broadcast over the entire specialty of alienism, or as they are pleased to dub these toilers in the service of humanity—the mad doctors—because of their enunciation of facts and opinions which their detractors have never had either the opportunity, the desire, or the courage to investigate, know assuredly but little of the true merits of the subject on which they so confidently pronounce judgment.

I have devoted a good deal of time, and of careful research, to the enquiry as to the present and past expressed opinions of asylum physicians on the vexed question of moral insanity; and I have found the views which close observance had early constrained me to entertain, so abundantly corroborated by a multitude of able writers and speakers, as to render retrogression from my position a very improbable, if not impracticable, movement.

In a very valuable work on the subject of moral insanity, published in 1870, by Dr. Bonfigli, of Ferrara, a concise review of the declared opinions of forty-six eminent alienistic writers on this subject is presented. These authorities may be divided as follows:

Seven, terminating with the epoch of Pritchard, uphold the doctrine of absolute, or pure and distinct, moral insanity; of these three were French, three German and one English.

Seventeen admit the term conditionally; that is to say, they recognize moral insanity as a conventional or convenient, but not as a distinct or pure form of mental disease. They hold that it is always associated with some degree of intellectual infirmity, or that it is the forerunner of insanity of the intellect. Of these seventeen, seven are French, six German, three Italian and one English.

Twenty-two absolulely, or impliedly, reject the doctrine in *toto*. Of these, nine are German, seven are French, five are Italian and one is American.

Had Dr. Bonfigli been more largely versed in the literature of English and American alienism, he could have much augmented the numbers assigned to the latter two countries; and, undoubtedly, the classes of conditional advocates and of utter repudiators would have had almost exclusive admission to his catalogue. He, however, introduces into his book a brief report of a discussion on moral insanity, which took place at the Annual Convention of Medical Superintendents of Asylums in New York, in the year 1863. I had the pleasure of being present and of taking part in this discussion, which was conducted in the most courteous and frank manner. Dr. McFarland gave it as his conviction that, "in all the cases of the so-called moral insanity, a real intellectual disorder was present." He was followed by the other members in rotation, including the distinguished and very long experienced Dr. Kirkbride, the President of the Association, and the veritable Nestor of the fraternity,—numbering in all present some forty representatives of the United States and Canadian Asylums. Of all this assemblage only two or three declared their belief in the actuality of moral insanity, and even these declined to define it as a distinct and independent form of the disease. Dr. Gray, Superintendent of the New York State Asylum, at Utica, said that in 5,000 cases of lunacy which had passed under his observance, he had not met with one of pure and distinct moral insanity. Dr. Chipley said he had not found one in 1,800 watched by him, and I made a similar statement as to 2,000 observed by myself. It is not, however, to be overlooked, that asylum physicians generally become first acquainted with the insane only after their malady has assumed a fully developed character. Very probably, had they more frequent opportunities of observing the disease in its incubative stage, they might feel inclined to recognize in it a *quasi* moral (or immoral) monopoly. Some sixteen years ago I encountered a case of ticketed moral insanity, sent to the Toronto Asylum by three respectable and and intelligent physicians. The subject was a girl of barely fifteen years. She was presented by her mother, who gave me a terrorizing history of the daughter's misdeeds, much of which I thought savored more of moral delinquency than of mental infirmity. However, she was sent to me as a lunatic, and I determined to treat her accordingly, regardless of all I had been told of her naughtiness. We began, as we ended, with uniform kindness. At the end of four and one-half months I wrote to her mother that she was either completely cured or she never had been insane. The mother was rejoiced to learn of the happy change, and she came promptly and took her daughter home; but on the second day after, she returned with her, and preseneed to me a large bag full of various articles of dress, on which Kate had been practicing dissections. I looked over them considerately, and on closing my inspection I said to the mother, "There is too much 'method in this madness' to convince me of its genuineness. We have had the girl here over four months, during which she has never spoken one word indicative of insanity, nor has she done one act pointing in that direction. I cannot re-admit her, for I believe she is not insane." Then I had a scene, which for long afterwards I did not understand, and, of course, could not justly appreciate. The distracted woman exclaimed, "Oh! what will become of her? She will go to the streets!" I then said, "Well, I will do this; I will give you the necessary blank forms of certificates of lunacy, and if you can get three physicians to sign them, I will take your daughter in again." And, as Colonel Prince once said, "it was done accordingly." So, back came my good girl, Kate, and I

gave her the benefit of a thirteen months' further probation, during all which she was just as good, as gentle, obedient and obliging as she had been throughout her former residence. I now talked to her in a very serious and paternal manner, showing her the impropriety and irrationality of her conduct at home, and pressing on her the consideration of her own best interests, which must be ruined by her continuance in a lunatic asylum. She listened to all I said with much deference, but finally told me she would like to leave the asylum, but not to go home to live with her mother. Now, her mother was neither harsh nor capricious, but, on the contrary, she had been both kind and forbearing; and her father and brothers had been equally so. I must say that this ultimate enunciation of my gentle patient let in a little light; for I well knew that the likings and dislikings of the insane are almost always unaccountable, and that both fall upon objects or persons apparently the most foreign to the rational incidence of either. I wrote to the mother, giving a faithful detail of all the facts, and advising the removal of her daughter from the asylum, but not her replacement in the family. She made suitable arrangements for the girl's residence at a distance in the country, and we had the pleasure of seeing her depart in excellent health, and in perfect mental composure. Three years afterward she paid us a visit, and I learned from her companion that she had shown no more symptoms of insanity, either moral or intellectual.

Now, suppose I had regarded and treated this young person, not as the subject of mental disease, but as a clear minded, moral delinquent; in other words, that I had, *quoad* her *exceptional* case, converted her asylum residence into prison correction; what would have been the probable result? It is my belief that I should then have transformed her into a real and a hardened criminal; or if there was, as I now verily believe there was, a constitutional strain of insanity in her frame, I should have been taking the shortest and surest course to precipitate its unmistakable development. Was it not worth while, even, to be deceived and imposed upon for the sake of this girl's rescue from a future of vice and misery? Hear me further before rendering your verdict.

Three or four years after parting with my grateful patient, a sister was brought to the asylum. There could be no question as to the reality of *her* lunacy. She was a sad wreck, both mentally and bodily. Some years before, she had left home and disappeared. No trace of her was had, until at last she was accidentally discovered as a demented inmate of a large pauper asylum in the United States. Her parents brought her home, and were soon obliged to bring her to me. When the mother now presented herself, and gave me the sorrowful history of this daughter's career, the echo of her distressful exclamation, when I had refused to re-admit her younger daughter, came back to my ears with thrilling accusation. But for the happy mental plasticity of the three medical gentlemen who certified to the moral insanity of my first patient, and thus secured her re-admission into the asylum, might not *she also* have fallen into a life of abandonment? Let him who will, answer the question, and then laugh at my ignorance as lustily and long as he pleases.

It is now my belief that my first patient was truly insane, call her insanity by what name soever you may choose; and I am convinced we took the only right course to prevent the more full development of her insanity, and to restore her to a state of intellectual and moral competency. Should I live long, I shall feel a deep interest in learning her future fortunes; for I by no means feel assured that she will come to old age without recurrence of her mental trouble.

Permit me here to introduce a case of flagitious criminality which occurred within the last few years, and came, as it manifestly deserved to do, under appropriate judicial censorship:

"Not long ago," says Dr. Clouston, "a lady, by a series of the most extraordinary misrepresentations and cleverly carried out impostures, raised large sums of money on no security whatever, and spent them as recklessly; imposed on jewelers, so that they trusted her with goods worth hundreds of pounds; furnished grand houses at the expense of trusting upholsterers; introduced herself by sheer impudence to one great nobleman after another, and then introduced her dupes, who, on the faith of these distinguished social connections, at once disgorged more money. To one person she was a great literary character; to another, of royal descent; to another, she had immense expectations; to another, she was a stern religionist."

This lady was, of course, finally brought to book. I leave to the fourth estate the measure of her punishment. She was an impostor, a huge liar, a cheat; she very well knew right from wrong, and transacted her business with great ability and skill. Not one of all those she duped and cheated—intelligent, prudent, and clear headed Scotchmen as they were—ever questioned her mental soundness. So we may readily conclude she was dealt with according to her demerits.

Let me complete, in the words of Dr. Clouston, Medical Superintendent of the Morningside Asylum, at Edinburgh, the history of this clever woman:

"At last all this lying, cheating, scheming and imposture developed into marked insanity and brain disease, of which she soon died; and it was seen that all these people had been the dupes of a lunatic, whose very boldness, cunning and mendacity had been the direct result of her insanity."

Yes, "it was seen that all these people had been the dupes of a lunatic." *When* was it so seen? Not assuredly whilst jewelers and upholsterers sold their goods to her on credit; not whilst noblemen admitted her into their select circle; nor whilst pious ministers regarded her as "a stern religionist." Had this poor woman's insanity not culminated speedily, but progressed slowly and insidiously, as it does in thousands of cases, she would, beyond all question, have been consigned to a penal prison; and had Dr. Clouston or any other physician ventured to express the opinion that she was insane when she committed the offenses charged against her, the judge would have frowned, the prosecuting counsel would have sneered, the jury would have been astounded, and the press would have applauded their verdict of *guilty*.

O! but we shall be told this woman did not commit murder. Well, let us be thankful for the accident; for who knows not how capricious, uncertain, and utterly outside the range of all the moral probabilities are the acts of the insane? *She* did not commit murder, because she was never tempted or provoked to do so; because she better attained her ends by milder means.

Her ends, however, were insane ends, and she *might*, dominated by a quickly-killing brain disease, have essayed their attainment by violent insane means. Poor thing! the only refuge to her, in escape from the barbarism of law and the blindness of justice, was the *madhouse!* How many a wretched victim of legal and judicial ignorance might, in a few years, or months, have found a similar refuge, had not the gallows anticipated the fiat of Nature?

[Dr. Workman now briefly related a few interesting details of two other cases of the so-called moral insanity, which came under his treatment in later years, both of which he regarded as genuine, though, as he frankly admitted, he had always failed to detect in either, whether in language or demeanor, anything so clearly indicative of intellectual defect, as might suffice to enable medical examiners to sign the certificate of lunacy requisite for their admission into an asylum. The statements, however, made by the friends of these patients, on which he had every reason implicity to rely, were of such a character as to convince him of the presence of actual insanity in both. It is, fortunately for asylum officers, a fact to them well known, that many of their patients behave while residents in asylums very differently from their conduct and language at home, and all that is necessary to re-develop their mental obliquity is to restore them to their former surroundings; many a family has had awful experience of this fact. In this country there is very little danger of persons in a sane state of mind being either committed to asylums or detained in them. No superintendent of any public asylum can have any interest whatever, in refusing to discharge a patient who has recovered for the credit side of his account in the public estimation, must consist mainly in the number of discharges of restored patients he is able to exhibit in his annual reports, so that whatever danger there may be in this relation, it must be rather on the side of liberating too many, than on that of detaining any wrongfully.]

Dr. Workman then said: "The subject to which I have to-night invited your attention is one that hardly falls within the usually recognized domain of practical medicine or surgery; yet I have but too frequently become cognizant of the fact that members of the profession have, sometimes very reluctantly, though in a few instances, rather exultingly, have called on to give testimony in cases involving the very important and often very obscure question of mental sanity or insanity; and I would be guilty of suppression of the truth, were I to withhold the expression of my constrained belief that the assurance with which some of these witnesses have renounced their opinions has ever been in the direct ratio of their ignorance of the general subject of insanity. As regards the very recondite question of moral insanity (so called), I have heard very loud denunciations of the term from men who had never read two pages either in affirmation or negation of the doctrine. It has been well said by some writer that nothing is so unanswerable as a sneer. Rely upon it, gentlemen, whenever you may have the misfortune, whether within or outside of the realm of insanity to appear in the witness box, the respect with which you will be heard will be in exact proportion to the extent of the knowledge of your subject possessed by your auditors; and too often this will not be very abundant, either on the bench, at the bar or in the jury boxes.

Before closing my remarks I would desire to allude to a difficulty in which medical witnesses are very liable to be involved, both within the courts of justice and outside of them. In cases of capital offenses, but more especially in those distinguished by great atrocity, as the crimes of the insane often are, the question will often be put to you, Why should such a criminal escape the gallows? Why should not he be held responsible to the law of the land? Now, I hold that with these questions the medical expert has nothing whatever to do. His function begins and ends with the simple establishment of the real mental state of the accused. If the law commands that whether sane or insane, he must be hanged, that should be none of your concern. If the law or its administrators, judging of his responsibility, not by his mental condition, but by the atrocity of the crime, send him to the gallows, the law and its administrators must bear the responsibility. And now Mr. President and gentlemen, in closing perhaps the last address I shall ever have the privilege of uttering in your presence, I would earnestly admonish you against ever in a court of justice using the term *moral insanity.—American Journal of Insanity.*

